# A web based dynamic MANA Nomogram for predicting the malignant cerebral edema in patients with large hemispheric infarction

**DOI:** 10.1186/s12883-020-01935-6

**Published:** 2020-09-29

**Authors:** Wenzhe Sun, Guo Li, Yang Song, Zhou Zhu, Zhaoxia Yang, Yuxi Chen, Jinfeng Miao, Xiaoyan Song, Yan Lan, Xiuli Qiu, Suiqiang Zhu, Yebin Fan

**Affiliations:** 1 Department of Neurology, Tongji Hospital, Tongji Medical College, Huazhong University of Science and Technology, No.1095 Jiefang Avenue, Wuhan, 430030 China; 2 School of Medicine and Health Management; Tongji Medical College, Huazhong University of Science and Technology, Wuhan, 430030 China; 3 Department of Radiology, Tongji Hospital, Tongji Medical College, Huazhong University of Science and Technology, No.1095 Jiefang Avenue, Wuhan, 430030 China; 4 The Solomon H. Snyder Department of Neuroscience, Johns Hopkins University School of Medicine, Baltimore, MD 21205 USA; 5 School of Computer Science and Technology, Huazhong University of Science and Technology, No.1095 Jiefang Avenue, Wuhan, 430030 China

**Keywords:** Ischemic stroke, Large hemispheric infarction, Brain edema, Nomogram, Atrial fibrillation

## Abstract

**Background:**

For large hemispheric infarction (LHI), malignant cerebral edema (MCE) is a life-threatening complication with a mortality rate approaching 80%. Establishing a convenient prediction model of MCE after LHI is vital for the rapid identification of high-risk patients as well as for a better understanding of the potential mechanism underlying MCE.

**Methods:**

One hundred forty-two consecutive patients with LHI within 24 h of onset between January 1, 2016 and August 31, 2019 were retrospectively reviewed. MCE was defined as patient death or received decompressive hemicraniectomy (DHC) with obvious mass effect (≥ 5 mm midline shift or Basal cistern effacement). Binary logistic regression was performed to identify independent predictors of MCE. Independent prognostic factors were incorporated to build a dynamic nomogram for MCE prediction.

**Results:**

After adjusting for confounders, four independent factors were identified, including previously known atrial fibrillation (KAF), midline shift (MLS), National Institutes of Health Stroke Scale (NIHSS) and anterior cerebral artery (ACA) territory involvement. To facilitate the nomogram use for clinicians, we used the “Dynnom” package to build a dynamic MANA (acronym for MLS, ACA territory involvement, NIHSS and KAF) nomogram on web (http://www.MANA-nom.com) to calculate the exact probability of developing MCE. The MANA nomogram’s C-statistic was up to 0.887 ± 0.041 and the AUC-ROC value in this cohort was 0.887 (95%CI, 0.828 ~ 0.934).

**Conclusions:**

Independent MCE predictors included KAF, MLS, NIHSS, and ACA territory involvement. The dynamic MANA nomogram is a convenient, practical and effective clinical decision-making tool for predicting MCE after LHI in Chinese patients.

## Background

Large hemispheric infarction (LHI) is a severe ischemic stroke involving a large portion of MCA territory with significant morbidity and mortality [1]. For LHI, malignant cerebral edema (MCE) is a life-threatening complication with a mortality rate approaching 80% [2, 3]. MCE is characterized by a malignant course of rapid neurological deterioration associated with massive cerebral swelling between the second and fifth day after stroke onset [4, 5], subsequent increased intracranial pressure (ICP), midline shift and brain herniation. To date, effective conservative treatment of MCE remains unsolved [6]. Moreover, treatment for MCE had largely focused on symptomatic patients rather than on edema prevention. Given the exceptionally high mortality rate associated with MCE, understanding the underlying mechanisms and predictors in patients with MCE, thereby enabling the identification of patients who will benefit from early intervention and providing effective approaches for preventing MCE, are important.

Previous clinical research has indicated demographic, clinical and radiographic predictors for MCE [7–10]. Reported predictors of MCE included younger age, higher National Institutes of Health Stroke Scale (NIHSS) [7], larger parenchymal hypoattenuation on computed tomography (CT) [11], hyperdense artery sign and higher blood glucose [10]. In recent years, diffusion weighted imaging (DWI) has been extensively used for MCE prediction [7, 9]. However, these indicators are not precise enough, not easy to obtain, or their predictive role in clinical settings is limited by their hysteresis [12]. Meanwhile, most known predictors are described in the Western context. Although China has experienced rapid health transitions over the last four decades, the lifetime risk of stroke in China is significantly higher than the global average [13]. With stroke being the first cause of death in China [14]. Hence, MCE in Chinese patients is worth exploring.

Furthermore, traditional MCE scoring models enrolled NHISS and MLS as categorical variables for its convenience by clinical use, which may end up degrading precision [15–18]. Nomograms can get rid of that limitation but are not as clinically accessible as scoring models. Motivated by the above facts, we sought to establish a web-based nomogram, with balanced precision and practicability, to forecast MCE in Chinese patients with LHI.

## Methods

### Patient selection

This study continuously enrolled 157 adult patients diagnosed with LHI and admitted within 24 h to the neuro-intensive care unit (NICU) of Tongji Hospital at Huazhong University of Science and Technology between January 2016 and August 2019. This retrospective study was approved by our Institutional Review Board and the need for written informed consent was waived. LHI was defined as infarction involving ≥50% of the territory of the middle cerebral artery (MCA) in computed tomography (CT) scan or DWI infarct volume > 82 mL within 24 h of onset [19]. Infarct volume was calculated using the ABC/2 formula [20]. MCE was defined as patient death or received DHC with obvious mass effect on follow-up imaging examination (≥ 5 mm midline shift or Basal cistern effacement on CT or DWI). Patients were selected for DHC at our institution based on the criteria outlined in previously published trials [21]. All patients completed the baseline CT scan or DWI at admission and had no primary intracranial hemorrhage. Other inclusion criteria for our study were: 1) Chinese ethnicity; 2) age ≥ 18 years; 3) first or recurrent acute stroke occurring within 24 h before admission. The exclusion criteria included: 1) Patients with terminal illness such as tumor, severe trauma, or other life-threatening diseases before admission; 2) patients without follow-up imaging examinations (CT scan or DWI) 24 h after onset; 3) death with secondary intracranial hemorrhage, acute myocardial infarction (AMI) or severe infection during hospitalization.

### Data collection

Admission characteristics were recorded for all patients, consisting of age, gender, smoking and drinking history, preexisting hypertension, diabetes mellitus, previous stroke, preexisting coronary heart disease, admission temperature, admission systolic pressure and admission diastolic pressure and previous atrial fibrillation (AF). AF was identified as previously known AF (KAF), differentiating from AF detected after stroke (AFDAS) [22]. Recorded treatments included intravenous thrombolysis or endovascular intervention. Laboratory tests on admission included baseline fasting blood glucose (FBG) and HbA1c. All 142 patients underwent CT/DWI within 24 h of onset and the imaging data were evaluated by two experienced clinicians, blinded to the patients’ outcome. Midline shift (MLS) was defined as the distance from the septum pellucida to the anatomic line anchored by the falx cerebri to the skull. Stroke severity was measured using the National Institutes of Health stroke scale (NIHSS). Cases without recorded NIHSS (*N* = 127, 89.4%) were not excluded from analysis. The NIHSS scores of all patients in this study were assessed by two experienced raters according to the patient’s admission documented neurologic exams. All raters were blinded to the patients’ outcome. An intra-rater reliability test was performed in 50 subjects, and the kappa values for MLS and NIHSS are 0.88 and 0.80, respectively.

### Statistical analysis

Statistical analyses were performed using IBM SPSS Statistics 22.0 (SPSS Inc., Chicago, IL, United States) and R version 3.5.2 (Institute for Statistics and Mathematics, Vienna, Austria; http://www.r-project.org/). Statistical analyses to identify risk factors were performed using SPSS version 22.0 (Statistical Package for the Social Sciences) for Windows (SPSS, Chicago, IL). Continuous variables (including MLS) are expressed as the median and interquartile range (IQR) while categorical variables as percentages. We performed binary logistic regression to determine predictors independently associated with MCE. Univariate analyses were conducted using univariate logistic regression analysis. Variables with *P* < 0.05 from the results of the univariate analyses were considered potential confounders and included in the multivariable model. The multivariable logistic regression using a backward stepwise method with input of variables if *p*-value < 0.05 and backward elimination if *p*-value > 0.05. All *P*-values were two-sided, *P* < 0.05 was considered statistically significant. Collinearity of variables that entered the multivariate logistic regression analysis was assessed by variation inflation factors (< 5 being considered nonsignificant) and tolerance (> 0.2 being considered nonsignificant).

The “rms” package and “Dynnom” package (cran.r-project. Org/web/packages/ rms) were used to construct a dynamic nomogram model. The performance of the dynamic nomogram was measured by the concordance index (C-index) and the area under the curve (AUC). The C-index was assessed by comparing nomogram-predicted probability versus observed probability. Bootstraps with 1000 resamples were applied to these activities. A higher C-index indicating better ability to separate patients with different MCE risk. Calibration curves were used to compare the predicted probability with the observed probability in the study. If the model calibration is correct, dots on the calibration plot should be close to a 45° diagonal line.

## Results

### Patient characteristics

In total, 142 patients with LHI were consecutively recruited in this study. Of all these potential subjects, 41 (28.9%) finally developed MCE (39 died, 2 received DHC). As shown in Table 1, patients with MCE were older (median, 64 vs. 58 years; mean, 65 vs. 58; *p* = 0.005), more likely to have preexisting coronary heart disease (24.4 vs. 8.9%; *p* = 0.018), previous stroke (31.7 vs. 15.8%; *p* = 0.037) or KAF (31.7 vs. 7.9%; *p* = 0.001), higher baseline NIHSS (median, 21 vs. 18; mean, 21 vs. 17; *p* < 0.001), higher baseline fasting blood glucose (median, 7.1 vs. 5.9 mmol/L; mean, 7.9 vs.6.4 mmol/L; *p* = 0.002) and greater admission MLS (median, 5.6 vs. 1.9 mm; mean, 6.2 vs. 3.7 mm; *p* < 0.001). In addition, patients with left hemisphere infarction (63.4 vs. 44.6%; *p* = 0.044) and patients with concurrent ACA (43.9 vs. 8.9%; *p* < 0.001), PCA (58.5 vs. 26.7%; *p* < 0.001) territory or basal ganglia (90.2 vs. 59.4%; *p* = 0.001) infarction were more likely to develop into MCE.

**Table 1:** Demographic and clinical characteristics of patients with and without MCE

Parameter	Non-ME (*N* = 101)	ME (*N* = 41)	*P*
Age, y, (IQR)	58 (51 ~ 67)	64 (57 ~ 71)	0.005*
Gender, male, N (%)	70 (69.3)	26 (63.4)	0.497
Smoke, N (%)	53 (52.5)	21 (51.2)	0.892
Drink, N (%)	44 (43.6)	14 (34.1)	0.302
Hypertension, N (%)	50 (49.5)	21 (51.2)	0.853
Diabetes mellitus, N (%)	18 (17.8)	9 (22)	0.570
Previous stroke, N (%)	16 (15.8)	13 (31.7)	0.037*
Preexisting coronary heart disease, N (%)	9 (8.9)	10 (24.4)	0.018*
KAF	8 (7.9)	13 (31.7)	0.001*
Treatment, N (%)			
Conservative (reference)	79 (78.2)	34 (82.9)	
Intravenous thrombolysis	16 (15.8)	4 (9.8)	0.362
Endovascular intervention	6 (5.9)	3 (7.3)	0.839
NIHSS (IQR)	18 (15 ~ 20)	21 (20 ~ 22)	< 0.001*
MLS, mm (IQR)	1.9 (3.6 ~ 5.1)	5.6 (4.9 ~ 8.1)	< 0.001*
Baseline temperature, °C (IQR)	36.5 (36.4 ~ 36.8)	36.5 (36.5 ~ 37.1)	0.170
ACA territory involvement, N (%)	9 (8.9)	18 (43.9)	< 0.001*
PCA territory involvement, N (%)	27 (26.7)	24 (58.5)	< 0.001*
Basal ganglia involvement, N (%)	60 (59.4)	37 (90.2)	0.001*
Cerebral hemisphere, right, N (%)	56 (55.4)	15 (36.6)	0.044*
Systolic pressure, mmHg, (IQR)	145 (127 ~ 166)	141 (120 ~ 159)	0.168
Diastolic pressure, mmHg, (IQR)	83 (73 ~ 95)	80 (74 ~ 92)	0.871
FBG, mmol/L, (IQR)	5.9 (5.4 ~ 6.8)	7.1 (5.8 ~ 9.2)	0.002*
HbA1c, %, (IQR)	5.6 (5.3 ~ 6.1)	5.7 (5.3 ~ 6.1)	0.378

*MCE*: Malignant cerebral edema, *IQR* Interquartile range, *KAF* Previously known atrial fibrillation, *NIHSS* National Institutes of Health stroke scale, *MLS* Midline shift, *ACA* Anterior cerebral artery, *PCA* Posterior cerebral artery, *FBG* Baseline fasting blood glucose, *HbA1c* Glycosylated hemoglobin

*p < 0.05 in univariate analysis were included in multivariable logistic regression models for adjustment

### Independent predictors and dynamic nomogram for predicting MCE

Variables showing *p* < 0.05 in univariate logistic regression were included in multivariable logistic regression models for adjustment (Table 1). No significant statistical collinearity was observed for these variables. After adjusting by potential confounders, KAF (aOR = 4.68, 95% CI, 1.42 ~ 15.42), MLS (aOR = 1.30, 95% CI, 1.04 ~ 1.62), NIHSS (aOR = 1.33, 95% CI, 1.07 ~ 1.66) and ACA territory involvement (aOR = 4.64, 95% CI, 1.59 ~ 13.60) were independent predictors (Table 2).

**Table 2:** Multivariate logistic regression model for MCE

Parameter	β	SE	*P*	OR (95%CI)
KAF	1.54	0.61	0.011*	4.68 (1.42 ~ 15.42)
MLS	0.26	0.11	0.023*	1.30 (1.04 ~ 1.62)
NIHSS	0.28	0.11	0.012*	1.33 (1.07 ~ 1.66)
ACA	1.54	0.55	0.005*	4.64 (1.59 ~ 13.60)

*MCE* Malignant cerebral edema, *KAF* Previously known atrial fibrillation, *NIHSS* National Institutes of Health stroke scale, *MLS* Midline shift, *ACA* Anterior cerebral arter, *OR* Odds Ratio, *CI* Confidence Interval, *SE* Standard Error

*Statistically significant at *p* < 0.05 level, two-sided

**Fig. 1:**
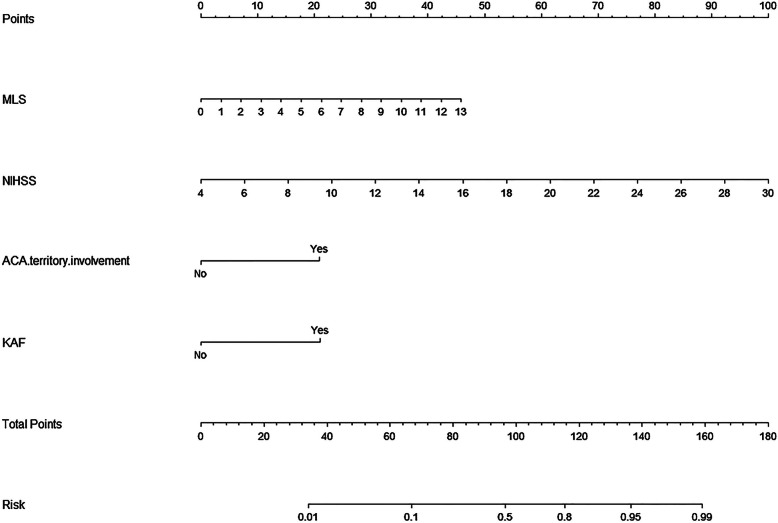
Nomogram for predicting malignant cerebral edema. The final score (i.e., total points) is calculated as the sum of the individual score of each of the 4 variables included in the nomogram. KAF: Previously known atrial fibrillation; NIHSS: National Institutes of Health stroke scale; MLS: Midline shift; ACA: Anterior cerebral artery

**Fig. 2:**
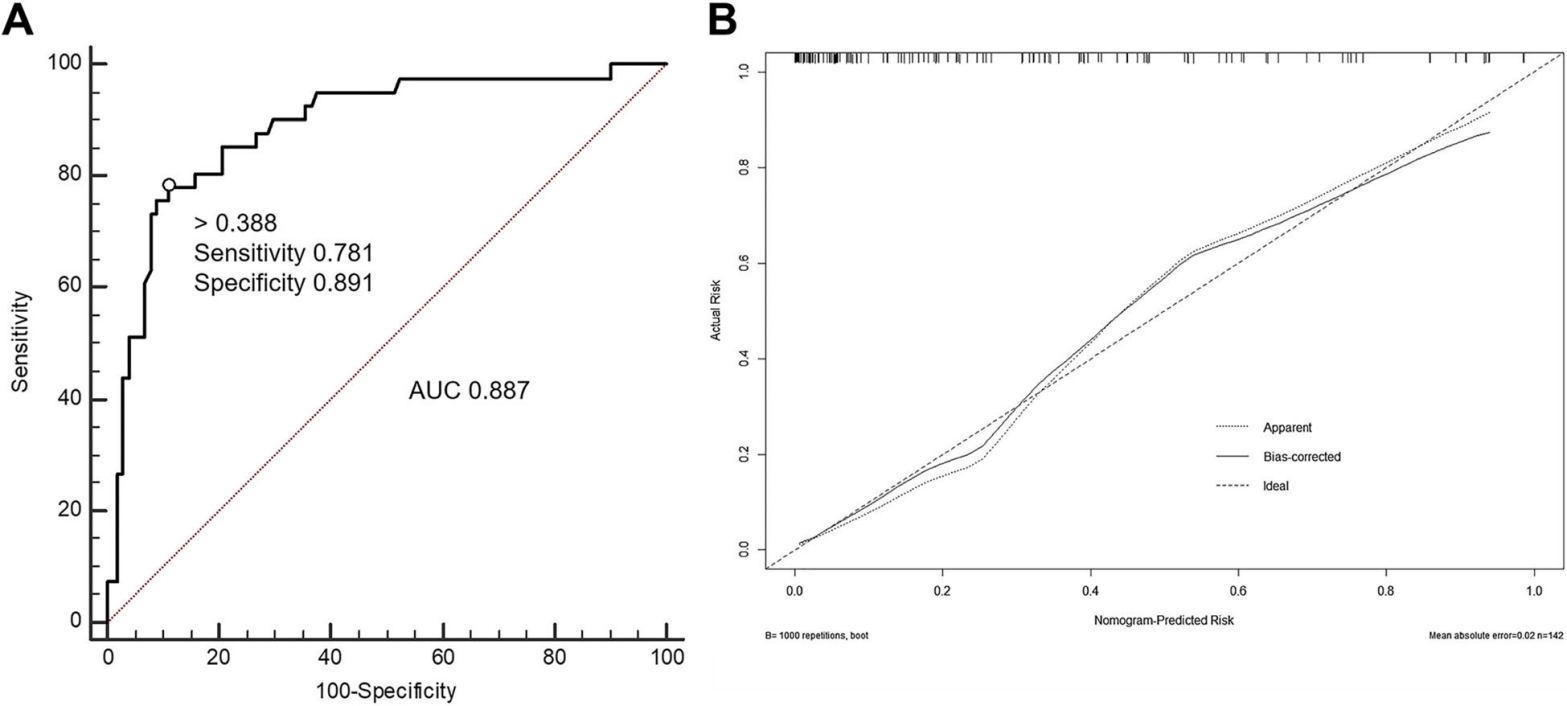
**a**: ROC curve of the nomogram used for predicting malignant cerebral edema in Chinese patients; **b**: Calibration curves for the nomogram used for predicting malignant cerebral edema. Dashed line is reference line where an ideal nomogram would lie. Dotted line is the performance of nomogram, while the solid line corrects for any bias in nomogram. AUC: Area under curve. ROC: receiver operating characteristic

**Fig. 3:**
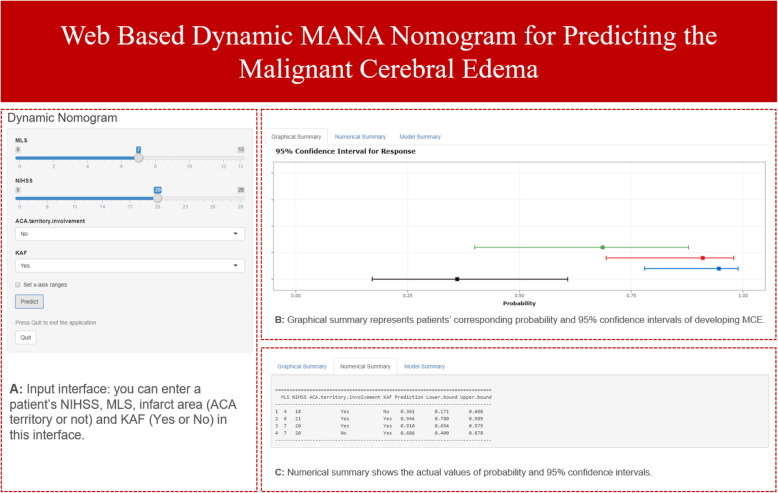
Operation interface of nomogram on web page. After entering a patient’s NIHSS, MLS, infarct area (ACA territory or not) and KAF (Yes or No) on http://www.MANA-nom.com, the neurologist can get the patient’s corresponding probability of developing MCE. **a**: Input interface, you can enter a patient’s NIHSS, MLS, infarct area (ACA territory or not) and KAF (Yes or No) in this interface. **b**: Graphical summary represents patients’ corresponding probability and 95% confidence intervals of developing MCE. **c**: Numerical summary shows the actual values of probability and 95% confidence intervals. MLS: Midline shift; NIHSS: National Institutes of Health stroke scale; ACA: Anterior cerebral artery; KAF: Previously known atrial fibrillation

All independent MCE predictors (Table 2) were considered to construct a nomogram (Fig. 1). This nomogram can predict MCE individually according to the various patient conditions. Each of the four independent predictors were projected upward to the value of the “Points” on top to get a score, with a point range from 0 to 100. Points assigned to the corresponding factors were summed to calculate the “total points”. The total score was then converted into an individual MCE risk. The higher the total score, the higher the risk of MCE. The predictive accuracy of the nomogram was validated using 1000 bootstrap samples, with a Harrell’s c-index value of 0.887 ± 0.041 (Fig. 2). The model was also internally validated in this cohort with an AUC-ROC value of 0.887 (95%CI, 0.828 ~ 0.934). Furthermore, to facilitate the use of the nomogram for clinicians, we used the “Dynnom” package to build an operation interface on a web page (www.MANAnom.com) to calculate the exact probability of developing MCE (Fig. 3). To help others to understand the MANA nomogram, we input four different combinations of the independent predictors, correspond to four imaginary patients. In Fig. 3, the four lines in different colors in part B corresponds to the predictions and 95% CI of these four patients. Part C shows these four patients’ actual numerical values of predictions and 95% CI.

## Discussions

We identified four independent MCE predictors in this study, Three of which consistent with previous studies, including NIHSS, MLS and ACA territory involvement [17, 18]. However the association between KAF and MCE is still controversial. On this basis we developed a visual MANA (MLS, ACA territory involvement and KAF) nomogram to assess the risk of MCE development of MCE in Chinese patients with LHI. The c-statistic of the MANA nomogram was up to 0.887 ± 0.041 while the AUC-ROC in this cohort was 0.887 (95%CI, 0.828 ~ 0.934).

For the past few years, with the development of cardiac monitoring technologies, physicians have noticed atrial fibrillation (AF) after ischemic stroke or transient ischemic attack (TIA). About 23.7% patients without AF before stroke later develop AF [23], termed AF detected after stroke (AFDAS). Nevertheless, the relationship between KAF and MCE has never been clarified. Currently, KAF is considered as the cardiogenic AF, which was mainly caused by cardiac remodeling, while AF detected after stroke (AFDAS) may be composed of multiple AF types, including preexisting but newly diagnosed atrial fibrillation (cardiogenic AF) and newly emerged atrial fibrillation (neurogenic AF) [24]. Neurogenic AF is the main type of AFDAS, which may be caused by the inflammatory response and dysfunction of the autonomic regulation of the cardiac rhythm [24, 25]. Based on this mechanistic difference between KAF and AFDAS, the effect of AFDAS on stroke severity may also vary from that of KAF. Previous research found that stroke patients with KAF have a higher rate of death or stroke recurrence (including hemorrhagic and ischemic stroke) than patients with AFDAS, but the difference was unadjusted [25]. To verify this supposition, we set up another multivariable regression to assess the differences in MCE risk among the sinus rhythm (SR), AFDAS and KAF (Table 3). After adjusting for confounders, we found that compared to patients with SR or AFDAS, patients with KAF had significantly higher risk of MCE (adjusted OR 4.29, 95% CI, 1.28 ~ 14.36). However, the risk between SR and AFDAS did not significantly differ. One possible reason is that patients with KAF had more severe hypoperfusion, leading to greater infarct growth and larger infarcts [26]. Our research also suggests that patients with KAF may have more severe stroke than patients with AFDAS (mean NIHSS 20 vs 18; mean infarct volume 231.1 mL vs 191.5 mL). Additionally, it is noticeable that risk factors of cardiac remodeling, such as endothelin-1 and matrix metalloproteinase, are also associated with brain edema [27–30]. Furthermore, neurogenic AF as “functional AF”, may have less AF burden than cardiogenic AF, which can also influence patient prognosis [31]. It is also worth noting that only 2.7% patients with AF in China received anticoagulant treatment [32], which may also be associated with the more severe ischemic stroke and brain edema incidence in China.

**Table 3:** Comparison of the risk of MCE among SR, AFDAS and KAF

Parameter	β	SE	*P*	OR (95%CI)
Rhythm				
SR (reference)				
AFDAS	−0.69	0.87	0.428	0.50 (0.09 ~ 2.77)
KAF	0.46	0.62	0.018*	4.29 (1.28 ~ 14.36)
MLS	0.25	0.11	0.026*	1.29 (1.03 ~ 1.61)
NIHSS	0.28	0.11	0.011*	1.33 (1.07 ~ 1.66)
ACA	1.63	0.57	0.004*	5.11 (1.68 ~ 15.54)

*MCE* Malignant cerebral edema, *SR* Sinus rhythm, *AFDAS* Atrial fibrillation detected after stroke, *KAF* Previously known atrial fibrillation, *NIHSS* National Institutes of Health stroke scale, *MLS* Midline shift, *ACA* Anterior cerebral artery, *OR* Odds Ratio, *CI* Confidence Interval, *SE* Standard Error

*Statistically significant at *p* < 0.05 level, two-sided

For patients with LHI, ACA territory involvement often hints the existence of larger infarction or more proximal vascular occlusion, such as carotid T or internal carotid artery (ICA) occlusion, less hemispheric collateral flow and greater volume of edematous brain tissue [15]. Further, the NIHSS score is correlated with stroke severity and infarct volume [10, 33], and MLS is a visual indicator on CT or MRI images, even sonographic monitoring, for assessing the severity of brain edema [34, 35]. Previous studies included NHISS and MLS as categorical variables into scoring models of malignant brain edema for the convenience of clinical use [10, 18]. However, compared to the continuous use of NIHSS and MLS, the categorical use loses precision. Despite the nomogram being able to circumvent that limitation, it has lesser practicability than scoring models. To make up for these deficiencies, we established a web operation interface (http://www.MANA-nom.com) for the MANA nomogram, which combines practicality and accuracy. Additionally, we did not collect data from CT angiography (CTA), DWI or special measurement techniques [17, 36–38], considering that the model needed to be available and propagable.

Unlike previous nomograms that roughly calculate an approximation, the dynamic MANA nomogram can provide an exact value. Furthermore, it’s convenient for neurologists all over the world. After entering a patient’s NIHSS, MLS, infarct area (ACA territory or not) and KAF (Yes or No) on http://www.MANA-nom.com, the neurologist can get the patient’s corresponding probability of developing MCE. The MANA nomogram can also be used to identify patients who need early surgical treatment, or aid in the decision-making process for patients with high likelihood of MCE of LHI.

## Limitations

This study has several limitations. First, since the data of this study was retrospectively collected from a single center in China, some information may not be accurate enough. Most patients didn’t have recorded NIHSS, and the calculated NHISS may have discrepancy with the actual situation. Second, distinguishing KAF and AFDAS through history and electrocardiograph (ECG) during hospitalization may not be sufficiently rigorous. Moreover, limited by current ECG monitoring technology, quite a few paroxysmal AF was undetected, which underestimated the number of patients with AFDAS. Third, defining the primary outcome as death with brain edema or received DHC might have made us neglect the fact that some patients developed severe brain edema but pulled through without DHC at discharge. In addition, this network prediction model sometimes leads to system crashes, which can be resolved by clicking “Quit” and re-logging in. Despite these limitations, our study provided a widely available prediction model for neurologists to assess the risk of MCE in patients with LHI.

## Conclusions

Our study aimed to finding the risk factors of MCE and establishing a convenient and accurate risk model to forecast MCE for Chinese patients with LHI. We found that KAF may increase the risk of MCE in patients with LHI. The dynamic MANA nomogram can help neurologists make clinical decisions and discuss prognosis to patients’ families. Additionally, further external validation through prospective, multi-center, large-scale trials of this model are also necessary.

## Data Availability

The datasets used and analysed during the current study are available from the corresponding author on reasonable request.

## Abbreviations

LHI: Large Hemispheric Infarction
MCE: Malignant cerebral edema
DHC: Decompressive hemicraniectomy
KAF: Previously known atrial fibrillation
MLS: Midline shift
NIHSS: National Institutes of Health Stroke Scale
ACA: Anterior cerebral artery
ICP: Intracranial pressure
CT: Computed tomography
DWI: Diffusion weighted image
NICU: Neuro-intensive care unit
MCA: Middle cerebral artery
IQR: Interquartile range
PCA: Posterior cerebral artery
FBG: Fasting blood glucose
HbA1c: Glycosylated hemoglobin
AF: Atrial fibrillation
AFDAS: AF detected after stroke
FBG: Fasting blood glucose
ICC: Intraclass correlation efficient
C-index: Concordance index
AUC: Area under the curve value
OR: Odds Ratio
CI: Confidence Interval
SE: Standard Error
TIA: Transient ischemic attack
ICA: Internal carotid artery
CTA: CT angiography
